# The toxin–antitoxin complex Fic‐1–AntF functions as a deAMPylase that regulates the activity of DNA gyrase

**DOI:** 10.1002/mlf2.70085

**Published:** 2026-06-25

**Authors:** Furong Chen, Liwei Guo, Canhua Lu, Wenjun Jiang, Zhao‐Qing Luo, Junfeng Liu, Li‐Qun Zhang

**Affiliations:** ^1^ State Key Laboratory of Agricultural and Forestry Biosecurity, MARA Key Lab of Pest Monitoring and Green Management, College of Plant Protection China Agricultural University Beijing China; ^2^ Department of Chemistry and Biology Liaocheng University Dongchang College Liaocheng China; ^3^ State Key Laboratory for Conservation and Utilization of Bio‐Resources in Yunnan Yunnan Agricultural University Kunming China; ^4^ Yunnan Academy of Tobacco Agricultural Sciences Kunming China; ^5^ Department of Respiratory Medicine, Center of Infectious Diseases and Pathogen Biology, Key Laboratory of Organ Regeneration and Transplantation of The Ministry of Education, State Key Laboratory for Diagnosis and Treatment of Severe Zoonotic Infectious Diseases The First Hospital of Jilin University Changchun China; ^6^ State Key Laboratory of Maize Bio‐breeding China Agricultural University Beijing China

**Keywords:** AMPylation, deAMPylation, Fic protein, post‐translational modification, toxin–antitoxin

## Abstract

Toxin–antitoxin (TA) systems found in diverse bacteria play important roles in their adaptation to changing environments. The toxin of the Fic‐1–AntF TA pair from *Pseudomonas bijieensis* strain 2P24 inhibits bacterial DNA replication by attacking the subunit B of DNA gyrase (GyrB) via AMPylation, while the antitoxin AntF blocks its enzymatic activity by forming a stable protein complex. Although many proteins, including bacterial toxins, have been found to catalyze AMPylation, few enzymes involved in reversing this modification have been described. In this study, we found that the Fic‐1–AntF complex functions as a deAMPylase to reverse GyrB modification imposed by Fic‐1. Structural and genetic analyses of the Fic‐1–AntF complex revealed that Glu28 of AntF is critical for catalysis. Thus, AntF not only functions to inhibit the activity of Fic‐1 but also cooperates with the toxin to return the modified substrate to its native form by de‐modification. Our results reveal a novel regulatory mechanism for bacterial toxin, which sheds light on the evolution of such enzymes, particularly those of multiple subunits.

## Impact statement

Bacterial toxin–antitoxin (TA) systems function to facilitate cell survival under stress conditions. Our previous study found that Fic‐1 of the Fic‐1–AntF TA system from *Pseudomonas bijieensis* strain 2P24 inhibits bacterial replication by inactivating the subunit B (GyrB) of DNA gyrase via AMPylation. One remaining issue in this system is whether and how the inhibition imposed by Fic‐1 is relieved. Here, we find that the Fic‐1–AntF complex is a deAMPylase that catalyzes the removal of the AMP moiety from modified GyrB. Our findings establish that a TA complex possesses a novel enzymatic activity to regulate the activity of the toxin, thereby addressing a longstanding question about the reversal of post‐translational modifications imposed by TA systems with protein AMPylation activity.

## INTRODUCTION

Bacterial toxin–antitoxin (TA) systems play important roles in bacterial physiology, particularly their survival under trophic stress, phage infection, and biofilm formation and regulation^1^, ^2^, ^3^, ^4^. Based on the structure and the mechanism of the antitoxin, TA is divided into eight types^4^. The toxins in type I to type VII TA systems are proteins, whereas in the type VIII system, the toxin is a small RNA^5^. In type I, type III, and type VIII TA systems, antitoxins are small noncoding RNAs; in contrast, antitoxins in type II, type IV, type V, type VI, and type VII TA systems are small proteins^6^. RNA‐type antitoxins modulate the activity of toxin proteins by inhibiting their translation (type I)^7^, ^8^ or by directly blocking the activity of the toxin (type III)^9^, ^10^. Proteinaceous antitoxins function either by forming a complex with the toxin (type II)^11^, ^12^ or by protecting the target without direct interaction with the toxin *per se* (type IV)^13^, ^14^. There are a few exceptions, including the antitoxin protein GhoS of type V, which functions as a sequence‐specific endonuclease to cleave mRNA of the toxin GhoT^15^, the antitoxin SocA of type VI, which serves as an adaptor protein that binds the toxin SocB to promote its degradation by ClpXp^16^, and the antitoxins of type VII, which inactivate their cognate toxins via post‐translational modifications (PTMs)^17^, ^18^. Both toxins and antitoxins in the type VIII system are RNAs, in which antitoxins regulate toxin activity by repressing its expression as an antisense RNA or by a mechanism similar to a CRISPR RNA^5^, ^19^.

Fic (filamentation induced by cyclic adenosine monophosphate) proteins are a family of enzymes that disrupt the function of their targets, mostly by AMPylation (also known as adenylylation)^20^. The Fic domain is a part of a larger Fido (Fic/Doc) superfamily^21^. These proteins contain the signature motif HXFX(D/E)GNGRXXR critical for catalysis^22^, and the activity of many of them is regulated by an inhibitory α‐helice (α_inh_), which harbors the conserved motif (S/T)XXXE(G/N)^23^. Fic enzymes are divided into three classes in accordance with the position of their α_inh_ elements. The Fic domain and the α_inh_ motif in class I reside in two separate proteins that often form a TA system. In classes II and III, the α_inh_ motif is embedded in the polypeptide of the Fic proteins, localizing in the amino and carboxyl portion of the Fic proteins, respectively^23^.

Fic‐1 is a class I Fic protein found in *Pseudomonas bijieensis* (formerly *P. fluorescens*) strain 2P24 that inhibits DNA replication, chromosome segregation, and cell division by AMPylating Y111 of GyrB^24^. The small α_inh_ protein AntF encoded by a gene immediately upstream of *fic‐1* interacts with Fic‐1 and inhibits its AMPylase activity. Apparently, Fic‐1 and AntF together form a TA system^24^. Other TA systems that use Fic protein as a toxin include YhfG/EcFicT of *Escherichia coli* and VbhA/VbhT of *Bartonella schoenbuchensis* ^23^, ^25^. Among these, VbhT interferes with bacterial growth by inactivating DNA gyrase and topoisomerase IV via AMPylation^26^. EcFicT^G55R^ causes cell filamentation at high cAMP concentrations and elevated temperature, but the underlying biochemical mechanism remains elusive^27^.

In addition to Fic proteins, AMPylation can be catalyzed by the GX_11_DXD motif found in proteins such as glutamine synthetase (GS)‐adenylyltransferase (ATase) of *E. coli* ^28^ and the multifunctional protein SidM (also known as DrrA) from the bacterial pathogen *Legionella pneumophila* ^29^, and by the pseudokinase fold associated with a large cohort of proteins utilized by diverse organisms, ranging from bacteria to mammals^30^. Recently, LnaB of *L. pneumophila* was found to transfer the AMP moiety from ATP onto the phosphoryl group of phosphoribosylated ubiquitin produced by members of the SidE family and the reversal enzymes DupA and DupB^31^, ^32^. The first identified deAMPylase is GS‐ATase, which removes the AMP moiety from glutamine synthetase^33^, ^34^. SidD of *L. pneumophila* functions to counteract the effect of SidM as a deAMPylase specific for modified Rab1, thus allowing the bacterium to temporally control the activity of the small GTPase Rab1 at different phases of infection^35^, ^36^, ^37^, ^38^. Recent studies in mammalian cells and *Drosophila* found that the class II Fic domain in the FICD (also known as HYPE) protein is able to catalyze both AMPylation and deAMPylation reactions on substrate proteins such as BiP, and the glutamate residue critical for the autoinhibitory activity is essential for the deAMPylase activity^39^, ^40^, ^41^. EfFic (class II) of *Enterococcus faecalis* is also capable of catalyzing both AMPylation and deAMPylation reactions, and the transition between the two activities is regulated by Mg^2+^ and Ca^2+^ ions^42^. These studies suggest that class II Fic proteins are bifunctional enzymes with opposite biochemical activities that distinctly regulate the function of their targets in response to internal or environmental cues. Yet, how the activity of class I Fic proteins is regulated is unknown. Here, we demonstrate that the Fic‐1–AntF complex is a deAMPylase that functions to remove the AMP moiety from modified GyrB.

## RESULTS

### DeAMPylation of modified GyrB in reactions containing AntF and Fic‐1

**Figure 1:**
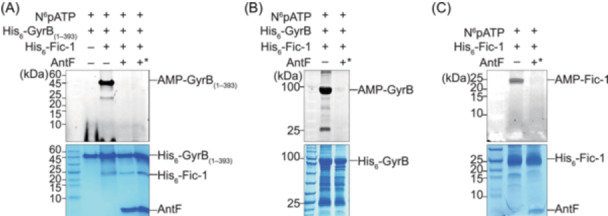
DeAMPylation of modified GyrB and Fic‐1 in reactions containing AntF and Fic‐1. (A) Fic‐1–AntF‐mediated deAMPylation of AMP‐GyrB. His_6_‐Fic‐1 and His_6_‐GyrB_(1–393)_ were incubated for 1 h without (lane 3) or with (lane 4) AntF. The asterisk indicates His_6_‐Fic‐1 that was incubated with His_6_‐GyrB_(1–393)_ for 1 h to produce N^6^pAMP‐GyrB _(1–393)_, followed by incubation with AntF for an additional 30 min (lane 5). (B, C) Fic‐1–AntF‐induced deAMPylation of AMPylated full‐length GyrB (B) and Fic‐1 (C). The reaction without AntF was incubated for 1 h (lane 2), and then AntF was added and incubated for another 30 min (lane 3). After sodium dodecyl sulfate‐polyacrylamide gel electrophoresis (SDS‐PAGE), N^6^pAMP‐GyrB (B) or N^6^pAMP‐ Fic‐1 (C) was detected by fluorescence signal measurement and proteins were detected by Coomassie brilliant blue (CBB) staining (lower panels).

In our experiments to determine the inhibition kinetics of AntF against Fic‐1, we added AntF to reactions in which His_6_‐Fic‐1 had been co‐incubated with His_6_‐GyrB_(1–393)_ and N^6^pATP for different durations. Incubation of His_6_‐Fic‐1 with His_6_‐GyrB_(1–393)_ and N^6^pATP for 1 h led to robust production of AMPylated GyrB_(1–393)_ (Figure 1A, lane 3). As expected, in reactions that received AntF and Fic‐1 simultaneously, no modified GyrB was detected (Figure 1A, lane 4). Interestingly, addition of AntF to samples containing AMP‐GyrB_(1–393)_ for 30 min rendered the modified product almost completely undetectable (Figure 1A, lane 5). Similar results were obtained when modified full‐length GyrB was used in the reaction (Figure 1B).

In the presence of ATP, Fic‐1 undergoes robust self‐AMPylation by modifying Y5, an event important for its enzymatic activity^24^. To examine whether the potential deAMPylation activity of the AntF also attacks modified Fic‐1, we added AntF to reactions containing AMP‐Fic‐1 for 30 min and found that modified Fic‐1 became undetectable in reactions receiving AntF (Figure 1C, lane 3). These results strongly suggest that in addition to its inhibitory activity against Fic‐1, AntF has deAMPylation activity toward both AMP‐GyrB and AMP‐Fic‐1. Because both full‐length GyrB and its deletion mutant GyrB_(1–393)_ can be indistinguishably AMPylated by Fic‐1, and the full‐length GyrB is less stable, creating noises that interfere with signal detection, we used GyrB_(1–393)_ in all of the subsequent experiments.

### The Fic‐1–AntF complex is a deAMPylase

**Figure 2:**
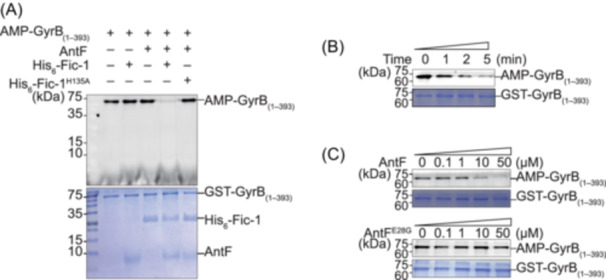
DeAMPylation of AMP‐GyrB requires both Fic‐1 and AntF. (A) Conditions required for deAMPylation of AMP‐GyrB_(1–393)_. Purified N^6^pAMP‐GyrB_(1–393)_ was used as a substrate, and the reaction mixtures containing AntF (lane 4), His_6_‐Fic‐1 (lane 3), or both (lane 5) were incubated for 30 min. Fic‐1^H135A^ is an AMPylation‐defective mutant. (B) Kinetics of the deAMPylation activity of Fic‐1–AntF. Reactions containing N^6^pAMP‐GyrB_(1–393)_, 50 μM AntF, and 50 μM His_6_‐Fic‐1 were initiated at 30°C, and samples collected at the indicated time intervals were analyzed for the modification as described in Figure 1. (C) Dose‐dependent deAMPylation by Fic‐1–AntF. Increasing concentrations of AntF were added to reactions containing His_6_‐Fic‐1 and N^6^pAMP‐GyrB_(1–393)_, and the mixtures were incubated at 30°C for 10 min. The inactive mutant AntF^E28G^ was included as a negative control. AMP‐GyrB_(1–393)_ was detected by fluorescence signal measurement (upper panels) and total proteins in the reactions were visualized by CBB staining (lower panels).

To discern the proteins required for the observed deAMPylation activity, we prepared AMP‐GyrB_(1–393)_ from reactions containing Fic‐1, GyrB_(1–393)_, and the ATP analog N^6^pATP (Figure 2A, lane 2). Incubation of AMP‐GyrB_(1–393)_ with AntF and Fic‐1 resulted in marked reduction of AMP‐GyrB_(1–393)_ (Figure 2A, lane 5), whereas the addition of the catalytically inactive mutant Fic‐1^H135A^ yielded no discernable decrease in signal intensity (Figure 2A, lane 6). In contrast, deAMPylation did not detectably occur in reactions that received only AntF or Fic‐1 (Figure 2A, lanes 3 and 4). These results indicate that Fic‐1 and AntF function together to catalyze the deAMPylation reaction. Since AntF forms a stable complex with Fic‐1 by direct protein–protein interactions^24^, we speculated that the Fic‐1–AntF complex functions as a deAMPylase. Consistent with this hypothesis, adding AntF and Fic‐1 simultaneously to reactions containing AMP‐GyrB_(1–393)_ quickly reduced the amount of modified GyrB_(1–393)_, with more than 50% modified protein being returned to the unmodified form within 2 min (Figure 2B). The effect of AntF was dose‐dependent because the rate of de‐modification increased when more protein was added (Figure 2C). Importantly, the mutant AntF^E28G^ defective in inhibiting Fic‐1 activity has lost the ability to deAMPylate AMP‐GyrB_(1–393)_ together with Fic‐1 (Figure 2C).

### The AMPylation and deAMPylation activities of Fic‐1 and the Fic‐1–AntF complex require specific metal ions

**Figure 3:**
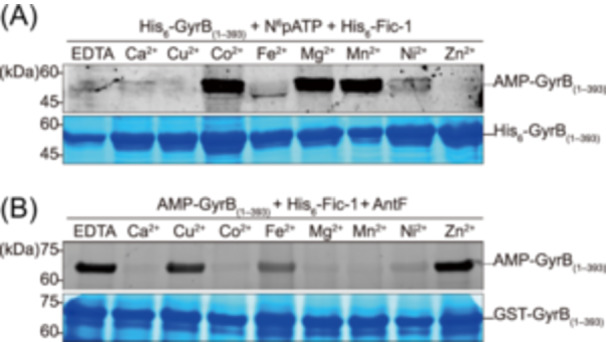
Impact of metal ions on the AMPylation and deAMPylation activities of Fic‐1 and its complex with AntF. (A) AMPylation reactions performed using N^6^pATP in samples supplemented with 2 mM of the indicated metal ions. (B) DeAMPylation of N^6^pAMP‐GyrB_(1–393)_ initiated by adding Fic‐1 and AntF to reaction mixtures containing 2 mM of the indicated metal ions. Reactions were incubated at 30°C for 10 min. N^6^pAMP‐GyrB_(1–393)_ was detected by fluorescence signal measurement (upper panels) and total proteins in the reactions were detected by CBB staining (lower panels).

Mg^2+^ and Ca^2+^ ions have been suggested to regulate AMPylation and deAMPylation activities of Fic enzymes^42^, we thus analyzed how divalent metal ions affect the activities of Fic‐1 and the Fic‐1–AntF complex. A series of divalent cations (2 mM) were tested in reactions designed to measure AMPylation or deAMPylation. Whereas Co^2+^, Mg^2+^, and Mn^2+^ each supported the AMPylation activity of Fic‐1 toward GyrB_(1–393)_ (Figure 3A), Ca^2+^, Co^2+^, Mg^2+^, Mn^2+^, or Ni^2+^ allowed the Fic‐1–AntF complex to deAMPylate AMP‐GyrB_(1–393)_ (Figure 3B). Thus, Mg^2+^, Mn^2+^, and Co^2+^ each supports both activities, and Ni^2+^ and Ca^2+^ only promote deAMPylation by the protein complex. When different concentrations of Ni^2+^ were tested, clear AMPylation activity of Fic‐1 was observed only at 10 mM, and not at lower concentrations (Figure S1). Given that the intracellular Ni²⁺ concentration in *P. bijieensis* 2P24 under physiological conditions is substantially lower than 10 mM, these results indicate that Ni^2+^ is unlikely to funciton as the physiologically relevant metal cofactor for Fic‐1‐AntF complex. In addition, Ca^2+^ could not inhibit the AMPylation activity of Fic‐1 in reactions containing Mg^2+^ (Figure S2), suggesting that Ca^2+^ is not the signal for the functional transition between AMPylation and deAMPylation.

### Formation of a stable protein complex by Fic‐1 and AntF is critical for the deAMPylation activity

**Figure 4:**
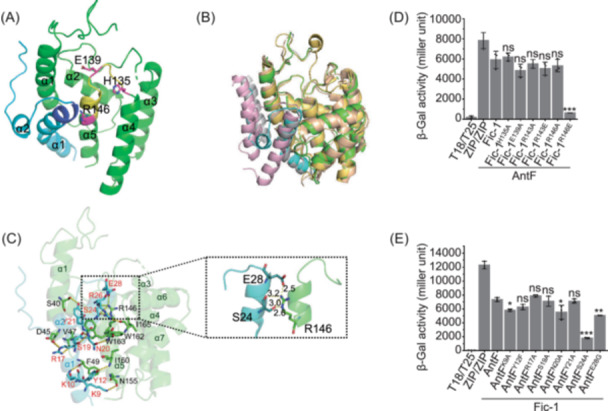
Structure of the Fic‐1–AntF complex. (A) Positioning of the Fic motif HPFX(D/E)GNGRXXR (X, any amino acid) within the structure. Fic‐1 is shown in green, with key catalytic residues highlighted in yellow. AntF is shown in light blue, with key catalytic residues highlighted in blue. (B) Structural comparison of the Fic‐1–AntF complex (green/light blue) with the EcFicT^G55R^/EcFicA complex (light orange/grey) from *Escherichia coli* (PDB: 5JFF) and of the VbhA/VbhT complex (yellow/pink) from *Bartonella schoenbuchensis* (PDB: 3SHG). (C) The Fic‐1–AntF complex structure depicted as ribbon diagrams, with Fic‐1 in green and AntF in cyan. Key interfacial residues are displayed as stick models, with carbon atoms colored according to the corresponding chain. Hydrogen bonds are represented by dashed lines. The critical interaction interface, formed by R146 of Fic‐1 and S24 and E28 of AntF through hydrogen bonds and slat bridges, is magnified in the inset. (D, E) Analysis of interactions between AntF and Fic‐1 mutants using bacterial two‐hybrid assays. Interaction strength was assessed by β‐galactosidase activity with ortho‐Nitrophenyl‐β‐galactoside (ONPG) as a substrate. The ZIP/ZIP interaction served as a positive control and strains harboring empty plasmids were used as negative controls. Data represent mean ± SEM from three independent experiments. Statistical significance relative to the wild‐type Fic‐1–AntF interaction was determined by one‐way ANOVA followed by Dunnett's post hoc test (ns, not significant; *p < 0.05; **p < 0.01; and ***p < 0.001).

Our biochemical results demonstrated that the Fic‐1–AntF complex functions as a deAMPylase to regulate the activity of GyrB. To investigate the catalytic mechanism of the deAMPylation reaction, we set out to determine the structures of Fic‐1 and the Fic‐1–AntF complex by X‐ray crystallography. By co‐expressing His_6_‐Fic‐1 and AntF in *E. coli*, we succeeded in obtaining crystals of the complex of the two proteins, which allowed us to solve its structure at a 2.5 Å resolution. The final refined model of the complex contained residues 10–197 of Fic‐1 and residues 5–47 of AntF (PDB code: 8WT0). Fic‐1 in the complex structure closely resembles the Fic domain structure in its apo form, both comprising of a bundle of seven α‐helices in which the Fic signature motif HXFX(D/E)GNGRXXR is situated between α4 and α5 (Figure 4A). This structure is highly similar to that of EcFicT^G55R^/EcFicA of *E. coli* (PDB code: 5JFF) and VbhA/VbhT of *B. schoenbuchensis* (PDB code: 3SHG), overlaying with an RMSD of 0.898 and 1.028 Å, respectively (Figure 4B). AntF forms a two‐helix anti‐parallel bundle that stably interact with Fic‐1 via hydrophobic contacts and 16 hydrogen bonds, predominantly between AntF and helices α1 and α5, α6 of Fic‐1 (Figures 4C and S3). The stability of the complex is maintained by an intimate hydrophobic interaction‐mediated binding interface that buries 18.42% and 48.96% of the solvent‐exposed surface area of the Fic‐1 (8365.70 Å^2^) and AntF (2997.03 Å^2^), respectively. Hydrogen bonds are distributed on four binding areas, including Fic‐1 residues on β2, β5, the loops between β2, β3, and AntF residues on β1. The most important binding area between Fic‐1 and AntF is formed by hydrogen bonding between S40, R146 of Fic‐1, and Y21, S24, and E28 of AntF. The second area involves W163, I165 of Fic‐1, and S19 and R26 of AntF. The third interaction site is centered on D45, V47, E48, I160, W162, and N155 of Fic‐1 and R17, Y12, N20, and K9 of AntF. The fourth area contains F49 of Fic‐1 and K10 of AntF (Figures 4C and S3). In agreement with the structural features, mutations in either R146 in Fic‐1 or S24 in AntF impaired the binding between these two proteins, as measured by bacterial two‐hybrid assays (Figures 4D,E and S4A,B). The importance of these residues in interactions between Fic‐1 and AntF was further corroborated by pull‐down experiments (Figure S4C,D).

**Figure 5:**
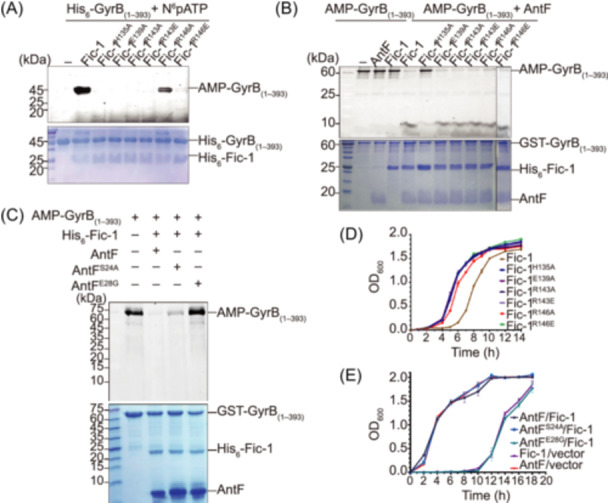
Identification of residues in Fic‐1 and AntF critical for deAMPylation activity. (A) AMPylation activity of Fic‐1 and its mutants. His_6_‐Fic‐1 or its mutants were added to reactions containing N^6^pATP and His_6_‐GyrB_(1–393)_. After incubation at 30°C for 10 min, samples were resolved by SDS‐PAGE and analyzed for AMPylated products. (B) DeAMPylation of AMP‐GyrB_(1–393)_ by AntF in combination with Fic‐1 mutants. Reactions containing AntF and AMP‐GyrB_(1–393)_ were supplemented with His_6_‐Fic‐1 or its mutants, and the remaining modified GyrB was detected after incubation at 30°C for 10 min. (C) DeAMPylation of N^6^pAMP‐GyrB_(1–393)_ by His_6_‐Fic‐1 in combination with AntF mutants. AntF or its mutants were added to reactions containing His_6_‐Fic‐1 and N^6^pAMP‐GyrB_(1–393),_ and after a 10‐min incubation at 30°C, samples were processed to determine the level of modification. In all panels, N^6^pAMP‐GyrB_(1–393)_ was detected by fluorescence signal measurement (upper panels) and total proteins were visualized by CBB staining (lower panels). (D, E) Cytotoxicity of Fic‐1 and its mutants in *E. coli*, and modulation of this toxicity by AntF and its mutants. Plasmids harboring *fic‐1* or its mutants were transformed into *E. coli* strain BL21(DE3), and bacterial growth in LB medium supplemented with IPTG was monitored (D). AntF or its mutants were individually co‐expressed in Fic‐1‐expressing strains, and bacterial growth was evaluated to assess functional interactions (E).

Our structure analysis reveals that E139, R143, and R146 of Fic‐1 and S24 of AntF are important for the formation of the Fic‐1–AntF complex (Figure 4A,C). In addition, superposition of our analyzed structure with the structure of the Fic domain of NmFic in complex with AMPPNP revealed that the conserved residues R139 and E143 in Fic‐1^H135A^ displayed marked deviations (Figure S5). We thus examined the role of these residues in the AMPylase and deAMPylase activities by creating alanine substitution mutants for each site and testing their biochemical activities. Fic‐1^R146A^, but not Fic‐1^E139A^ and Fic‐1^R143A^, remained active in AMPylation (Figure 5A). In contrast, Fic‐1^H135A^ was the only Fic‐1 mutant that had lost deAMPylase activity after forming a complex with AntF (Figures 5B and 2A). In the Fic‐1–AntF complex, the side chain of R146 of Fic‐1 forms a hydrogen bond with the side chain of S24 of AntF; it also forms a salt bridge with the side chain of E28 of AntF (Figure 4C). Whereas the AntF^E28G^ mutant lost the deAMPylase activity, the AntF^S24A^ mutant partially retained the ability to deAMPylate together with Fic‐1 despite the fact that this mutant had largely lost the ability to bind Fic‐1 (Figures 4E and 5C).

Consistent with their enzymatic activity, only mutant Fic‐1^R146A^ displayed detectable toxicity to *E. coli*; other mutants including Fic‐1^H135A^, Fic‐1^E139A^, Fic‐1^R143A^, Fic‐1^R143E^, and Fic‐1^R146E^ had completely lost such toxicity (Figure 5D). Furthermore, mutant AntF^S24A^ inhibited the activity of Fic‐1 at a level comparable to that of wild‐type protein. In contrast, AntF^E28G^ could no longer inhibit the toxicity of Fic‐1 to *E. coli* (Figure 5E). Taken together, these results demonstrate that while H135 of Fic‐1 is essential for both AMPylation and deAMPylation activities, E139, R143, and R146 are specifically required for AMPylation activity. On the AntF side, E28 is critical for both the inhibitory effect against Fic‐1 and the deAMPylase activity.

### A negatively charged cleft centered by E28 of AntF is essential for deAMPylation activity

**Figure 6:**
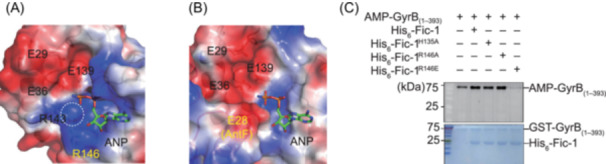
A negatively charged cleft surrounding the active site of the Fic domain is essential for deAMPylation activity. (A,B) Molecular electrostatic surface potentials of Fic‐1^H135A^ (A) and the Fic‐1–AntF complex (B). Positively and negatively charged regions are represented in blue and red, respectively. The position of the γ‐phosphate of ANP/ATP is indicated by a white circle (A); AntF is shown in purple (B). (C) DeAMPylation of N^6^pAMP‐GyrB_(1–393)_ by Fic‐1 mutants. His_6_‐Fic‐1 or its mutants were added to reactions containing N^6^pAMP‐GyrB_(1–393)._ After incubation at 30°C for 10 min, samples were processed to detect AMP‐GyrB. N^6^pAMP‐GyrB_(1–393)_ was detected by fluorescence signal measurement (upper panel), and total proteins were visualized by CBB staining (lower panel).

Despite extensive efforts, we were unable to obtain structures of the Fic‐1–AntF complex that contained ATP or AMP; we thus attempted to position the ATP analog AMPPNP in the structure of Fic‐1^H135A^ and the Fic‐1–AntF complex by aligning the three key residues (H…D/E…R) of Fic motifs known to be involved in binding the nucleotide in the structure of the NmFic/AMPPNP complex (PDB code: 3S6A). A distinct distribution of surface charges was observed. For Fic‐1^H135A^, the γ‐phosphate of ANP/ATP was located in the conserved Fic domain with a positively charged patch (Figure 6A), but for the Fic‐1–AntF complex, the γ‐phosphate was exposed to the negatively charged patch formed by E29, E36, and E139 of Fic‐1 and E28 of AntF (Figure 6B). Because only the side chain of R146 of Fic‐1 contributes to the interaction with the side chains of S24 and E28 of AntF, we speculated that Fic‐1^R146^ and AntF^E28^ are responsible for altering the charge status of the patch, resulting in the functional switch from AMPylation to deAMPylation. Consistent with this hypothesis, mutant Fic‐1^R146E^, but not Fic‐1^R146A^, retained the deAMPylation activity even in the absence of AntF (Figure 6C). In the presence of AntF, both Fic‐1 and its R146E mutant showed comparable time‐dependent deAMPylation activity (Figure S6). These findings suggest that the negatively charged side chain of Glu provided by either AntF^E28^ or Fic‐1^R146E^ is sufficient to catalyze the deAMPylation reaction.

## DISCUSSION

The reversal of protein PTM allows cells to precisely modulate signaling cascades in response to changes in external or internal cues. Like other PTMs, signaling by protein AMPylation is regulated by specific enzymes. For example, AMPylation of the small GTPase Rab1 induced by SidM is reversed by SidD, which allows GTP hydrolysis to occur and the removal of Rab1 from the *L. pneumophila* phagosome^35^, ^37^. Similarly, class II Fic proteins, such as FICD and EfFIC, function as both AMPylase and deAMPylase, a switch that is regulated by cell homeostasis and fluctuations in the cellular concentration of Mg^2+^ ^42^. A recent discovery reveals that ribonuclease RNase Z functions as a deAMPylase, specifically catalyzing the removal of AMP from protein substrates that have undergone AMPylation mediated by the pseudokinase Selenoprotein O (SelO)^43^. Our results here demonstrate that the protein complex formed by a class I Fic protein and its inhibitory protein function as a deAMPylase to reverse the modification imposed by the Fic protein. This finding solves the mysterious absence of a deAMPylase associated with class I Fic proteins. Under normal physiological conditions, it is believed that class I Fic proteins such as Fic‐1 are in complex with their inhibitor and they will be freed to induce AMPylation on their target under stress conditions that lead to the dissociation of the complex^24^. Once the stress is relieved, the abundance of the inhibitory protein will increase, leading to the arrest of the AMPylation activity. Our results suggest that modified GyrB will be returned to its native status by the toxin–antitoxin complex, thus allowing DNA replication and cell division to resume (Figure S7). Comparing with TA systems in which the substrate protein needs to be resynthesized after the damaging condition has been eliminated, the de‐modification mechanism used by Fic‐1 and AntF clearly will allow the bacteria to resume normal cellular process more efficiently with less energy expenditure.

The Fic‐1–AntF TA pair shares structural and mechanistic similarities with type VII TA systems; yet, it also shows distinct functional differences. In type VII systems, both the toxin and the antitoxin are proteins, and the antitoxin neutralizes toxin activity through PTM^18^. Recent studies revealed that type VII systems MenA_1_T_1_ and MenA_3_T_3_ (TakA/TglT) in *Mycobacterium tuberculosis* use a distinctive detoxification strategy^17^, ^44^. Although antitoxins MenA_1_ and MenA_3_ lack canonical kinase motifs, these proteins interact with the nucleotidyltransferases (NTase) MenT_1_ and MenT_3_, respectively, and induce auto‐phosphorylation at critical residues (T39 in MenT_1_ and S78 in MenT_3_), thereby effectively inhibiting the NTase activity^44^. A notable functional parallel between the Fic‐1–AntF and MenAT systems is that both TA complexes acquire a novel enzymatic activity after forming protein complexes. Despite these similarities, the two systems differ significantly in their molecular targets: whereas the MenAT complex suppresses toxicity via phosphorylation of the toxin, the Fic‐1–AntF complex catalyzes deAMPylation of target proteins, reversing the inhibitory effect imposed by PTM.

Phylogenetic analysis suggests that class I Fic proteins are originated from class II Fic proteins (α_inh_ in the N‐terminal)^45^. Gene fusion/fission is considered the major mechanism of the evolution of multi‐domain proteins^46^. Gene fission events create independently expressing proteins that harbor the required domains; these may provide class I Fic proteins such as Fic‐1–AntF more flexibility in functionality and more diverse mechanisms to cooperate with other proteins to respond to more complex cues.

The mutant AntF^S24A^ was severely impaired in its interaction with Fic‐1 (Figure 4E), but had retained considerable deAMPylation activity (Figure 5C). A possible explanation for this observation was the excessive AntF^S24A^ used in our biochemical experiments. The high protein abundance may have satisfied the requirement for productive interactions (Figure 5C). This notion was supported by the concentration gradient test, in which Fic‐1‐AntF^S24A^ showed significantly lower deAMPylation activity compared to Fic‐1–AntF at the same concentrations (Figure S8).

Structure‐guided simulation revealed the exposure of the γ‐phosphate of AMPPNP to the negatively charged environment contributed by AntF (Figure 6B), and our genetic evidence further demonstrated the critical role of E28 in AntF for deAMPylation (Figure 6C). These findings support the conclusion that E28 contributes to the formation of a negatively charged region within the Fic‐1–AntF complex that is essential for deAMPylation. Similarly, a recent study has shown that the deAMPylation activity of the *E. faecalis* Fic protein (EfFic) requires a glutamate residue homologous to E28 in AntF^42^. In human FICD, E234, previously identified as a key residue in inhibiting the AMPylation reaction, has also been considered essential for deAMPylation^41^. However, recent theoretical simulations indicate that the free‐energy barrier of deAMPylation is nearly identical between the wild‐type enzyme (~17.3 kcal/mol) and the E234A mutant (~17.1 kcal/mol), suggesting that E234 does not directly affect the deAMPylation reaction^47^. This implies functional divergence of this conserved glutamate residue in mammalian orthologs, where its role in catalysis may be less constrained compared to bacterial systems.

Surface charge simulations of Fic‐1^R146E^ in the presence of AMPPNP revealed a marked electronegative surface region, particularly when compared to the Fic‐1^H135A^, which has completely lost the activity (Figure S9A). In contrast, this region was positively charged in Fic‐1–AntF^E28G^ (Figure S9B). Thus, the negative charge provided by the side chain of a glutamate residue in this region by AntF^E28^ or Fic‐1^R146E^ is critical for the deAMPylation activity. Interestingly, examination of 65,536 FIC/DOC proteins from UniProt revealed that at least 95 proteins harbor such a signature, including the BepA‐like protein of *Gluconobacter cerinus* (Figure S10). We were unable to test the activity of these proteins due to the lack of the modified substrates. Whether these proteins indeed have de‐modification activity in the absence of their inhibitory protein, if any, remains to be investigated.

In summary, the Fic‐1–AntF complex from *P. bijieensis* 2P24 functions as a dedicated deAMPylase that reverses Fic‐1‐mediated AMPylation of GyrB. This activity strictly depends on AntF's E28 residue and the formation of a stable Fic‐1–AntF complex. AntF plays a dual regulatory role: it suppresses Fic‐1's intrinsic AMPylation activity and, through direct complex formation, enables cooperative deAMPylation of modified GyrB. These findings reveal a previously unrecognized regulatory mechanism of class I Fic proteins and expand the functional repertoire of bacterial toxin–antitoxin systems in mediating reversible PTMs.

## MATERIALS AND METHODS

### Bacterial strains, plasmids, and media

*E. coli* DH5α and *E. coli* BL21 (DE3) were grown in Luria–Bertani (LB) medium at 37°C; *P. bijieensis* 2P24^48^, and *E. coli* BTH101^49^ were grown in LB medium at 28°C (Table S1). Antibiotics were added to the media at the indicated concentrations as needed: ampicillin at 50 µg/ml, kanamycin at 50 µg/ml, chloramphenicol at 20 µg/ml, and tetracycline at 10 µg/ml.

### Bacterial two‐hybrid assays

The Cya‐based bacterial two‐hybrid system^49^ was used to examine interactions of proteins*. fic‐1* was cloned into pKT25 and the *antF* or the *antF* mutant was cloned into pUT18C, or *antF* was cloned into pKT25, and the *fic‐1* or *fic‐1* mutant was cloned into pUT18C. Plasmid pairs were introduced into *E. coli* BTH101 and the interaction between Fic‐1 and AntF was probed on LB plates supplemented with X‐gal. The intensity of cellular interactions was evaluated quantitatively by measuring β‐galactosidase activity. Cells (100 μl) from saturated cultures were subjected to ONPG‐based enzymatic assays, with all operations carried out following the standardized experimental protocol^50^.

### Site‐directed mutagenesis

We used PCR to introduce mutations into specific sites of genes of interest by the high‐fidelity PrimeSTAR® Max DNA Polymerase (TaKaRa). The primers were designed by the QuikChange® Primer Design Program (Agilent Technologies). All mutations were verified by double‐strand DNA sequencing.

### Expression and purification of recombinant proteins

Unless otherwise specified, recombinant proteins were expressed in *E. coli* strain BL21 (DE3). The bacterial culture was inoculated into 250 ml of LB broth supplemented with appropriate antibiotics and cultured with shaking at 200 rpm, until the OD₆₀₀ reached 0.6–0.8 (for Fic‐1 expression, the OD₆₀₀ was maintained between 0.4 and 0.5). After adding IPTG to a final concentration of 1 mM, incubation was continued at 18°C in a shaker (200 rpm) for 16 h. The cells harvested by centrifugation at 4000*g* for 15 min were resuspended in a cold lysis buffer (20 mM Tris‐HCl pH 8.0, 150 mM NaCl, 10 mM imidazole, 5 μM benzamidine, and 1 μM phenylmethanesulfonyl fluoride [PMSF]) and were lysed by sonication. Intact cells and cellular debris were removed by sequential centrifugation at 12,000*g* for 15 min at 4°C, repeated twice. The resulting supernatant was incubated with 1 ml of Ni^2+^–NTA agarose beads in Falcon tubes for 2–3 h at 4°C to allow target protein binding. Subsequently, the bead mixture was packed into a 30 ml chromatography column and washed with lysis buffer equivalent to 10x the bed volume. Proteins eluted with an elution buffer (20 mM Tris‐HCl pH 8.0, 150 mM NaCl, and 250 mM imidazole) were dialyzed in a dialysis buffer (20 mM Tris‐HCl pH 8.0, 150 mM NaCl, 20% (v/v) glycerol, and 1 mM β‐mercaptoethanol).

### Fmoc‐based solid‐phase peptide synthesis (SPPS)

AntF, AntF^S24A^, and AntF^E28G^ used in the deAMPylation assays were synthesized by Fmoc‐based SPPS and purified by reversed‐phase high‐performance liquid chromatography (RP‐HPLC) as previously described^51^. All peptides were synthesized using the LibertyBLUE peptide synthesizer (CEM). All peptides with C‐terminal carboxylate were elongated on Fmoc‐Thr(tBu)‐Wang resin. For each carboxylate peptide, the first residue (Thr) was manually attached to the resin by dissolving 4 equivalents (equiv) Thr and 3.8 equiv O‐(6‐chloro‐1H‐benzotriazol‐1‐yl)‐N,N,N',N'‐tetramethyluronium (HCTU) in N,N‐dimethylformamide (DMF), followed by activation with 8 equiv N,N‐diisopropylethylamine (DIEA) for 30 s, before addition to the Wang resin for 1 h. The resin was then washed with DMF and coupled overnight (more than 12 h) by dissolving 4 equiv Thr and 4 equiv Oxyma in DMF, followed by activation with 8 equiv DIC for 10 min before addition to the Wang resin. The resin was then washed with DMF, and unreacted sites were capped by treatment with 1:8:1 acetic anhydride:DMF:DIEA twice (7 and 10 min, respectively). All resins were swelled in dichloromethan (DCM)/DMF for 30 min before deprotection. Fmoc‐protecting groups on both the resin and coupled amino acids were removed by treatment with DMF containing 20% piperidine and 0.1 M Oxyma for 2 min at 88°C. Prior to each deprotection step and subsequent condensation reaction, the resin was thoroughly washed with DMF and DCM. Automated peptide synthesis was performed at 88°C for 3 min using 4 equiv Fmoc‐amino acids, 4 equiv Oxyma, and 8 equiv DIC. Following the assembly of all amino acids, the residual Fmoc group was cleaved, and the resin was treated with a cleavage cocktail (trifluoroacetic acid [TFA]/H₂O/TIPS/thioanisole/EDT, 82.5/5/5/5/2.5) for 3 h at 30°C under gentle agitation. A major portion of the TFA was then removed by nitrogen blowing, and cold diethyl ether was added to precipitate the crude peptide. Subsequent to centrifugation, the supernatant was discarded, and the obtained precipitates were washed twice with diethyl ether. The crude peptides were solubilized in CH_3_CN/H_2_O, subjected to analysis by RP‐HPLC and ESI‐MS, and purified via semi‐preparative RP‐HPLC (Figure S11).

### Protein crystallization

The His_6_–Fic‐1–AntF complex and the Fic‐1^H135A^ mutant were crystallized at 291 K using the sitting‐drop diffusion method in MRC 96‐well plates (XtalQuest Ltd.) with an Oryx4 crystallization robot (Douglas Instruments) as described^52^. The His_6_–Fic‐1–AntF complex was concentrated to 9.7 mg/ml in a buffer containing 20 mM Tris–HCl (pH 8.0) and 150 mM NaCl. Drops with a volume of 0.5 μl were prepared by diluting the protein complex solution to various concentrations using the reservoir solution and then equilibrated against 35 μl of well solution. The crystallization conditions for the complex were randomly screened using the PEG/ION kit from Hampton research (http://www.hamptonresearch.com), the MCSG‐1, MCSG‐2, MCSG‐3, and MCSG‐4 screens from Microlytic (http://www.microlytic.com) as well as the Bioxtal crystallization kit from XtalQuest (http://www.xtalquest.com/default.aspx). After 8 days, small crystals of His_6_–Fic‐1–AntF appeared in conditions of 2 M Li_2_SO_4_, 0.05 M MES (pH 5.6), and 0.01 M MgCl_2_. The reservoir solutions were further optimized by using grid screening around the original condition. High‐quality crystals were obtained from 1.5 M Li_2_SO_4_, 0.1 M MES (pH 5.6), and 0.01 M MgCl_2_ within 10 days.

Crystals of the Fic‐1^H135A^ mutant were grown using a purified protein concentrated to 5.27 mg/ml by adding 5 mM AMPPNP. High‐quality crystals were obtained within 3–5 days using a reservoir solution containing 0.2 M magnesium formate dihydrate and 20% (w/v) PEG3350.

### Data collection and structure determination

Prior to data collection, crystals were transferred into the reservoir solution with additional 20% glycerol as a cryo‐protectant, mounted into a loop, and flash‐cooled in liquid nitrogen for later use. Native and single‐wavelength diffraction X‐ray datasets were collected at 100 K on beamline BL‐18U at the Shanghai Synchrotron Research Facility (SSRF). Data were indexed and scaled with xia2, which is implemented in the CCP4 software suite.

The initial structure was built using the molecular replacement (MR) method^53^ with Phaser using the previously determined VbhT/VbhA (PDB entry: 3SHG) and EcFicT/EcFicA (PDB entry: 5JFF) structures as templates. The final model was subsequently improved through iterative rounds of manual rebuilding and refinement using COOT combined with TLS (Translation/Libration/Screw) and PHENIX refine. The final refinement statistics are given in Table S2.

Stereochemical validation of the model was performed using MolProbity. The atomic coordinates and structure factors for the Fic‐1–AntF complex have been deposited in the Protein Data Bank under the accession code 8WT0. All structural figures were generated with PyMol (The PyMol Molecular Graphics system, Version 1.3 Schrodinger, LLG) and the protein–protein interface was analyzed using LigPlot^+^.

### Biochemical AMPylation assays

The AMPylation assay was performed as previously described^54^, with minor modifications. Briefly, 3 µg of purified Fic‐1 was incubated with 10 µg of recombinant His_6_‐GyrB or GST‐GyrB for 1 h at 30°C in a 20 μl reaction buffer (25 mM Tris‐HCl, pH 7.5, 50 mM NaCl, 3 mM MgCl_2_, 0.5 mM EDTA, and 100 μM N^6^pATP). The AMPylation reaction was stopped by methanol/chloroform precipitation. To a 15‐µl reaction mixture, 85 µl of water, 400 µl of methanol, and 100 µl of chloroform were added, and the samples were vortexed. Subsequently, an additional 300 µl of water was added, followed by vigorous vortexing. The samples were subsequently centrifuged at 12,000*g* for 5 min at room temperature. The upper phase was discarded, and 1 ml of ice‐cold acetone was added to the pellets. The samples were vortexed thoroughly and then centrifuged at 12,000*g* for 15 min at 4°C. The supernatant was discarded and protein pellets were dried for 5 min. The dried pellets were then solubilized in 15 µl of 4ST buffer (4% SDS, 150 mM NaCl, and 50 mM triethanolamine, pH 7.5). To each sample, 10 µl of click‐chemistry master mix (0.1 mM az‐rho, 1 mM TCEP (Tris(2‐carboxyethyl) phosphine), 0.1 mM TBTA (Tris[(1‐benzyl‐1H‐1,2,3‐triazol‐4‐yl)methyl]amine), and 1 mM CuSO₄) was added to bring the total volume to 25 µl. The click‐chemistry reaction was incubated at 30°C for 1 h and subsequently terminated by the addition of 8.3 µl of 4× Laemmli buffer. Samples were incubated at 95°C for 5 min and analyzed by 10% sodium dodecyl sulfate‐polyacrylamide gel electrophoresis (SDS‐PAGE). Protein gels were scanned on Azure Biosystems C600. All image adjustments were carried out on the entire depicted image for all samples equally in Adobe Photoshop CS3. Following fluorescence detection, protein gels were stained with Coomassie brilliant blue (BioRad).

### Biochemical deAMPylation assays

Fic‐1 was incubated with GST‐GyrB_(1–393)_ for 1 h at 30°C in 20 μl of AMPylation buffer. Ten microliters of GST beads was added to the AMPylation reaction and incubated for 1 h. The beads were washed 5 times with washing buffer (25 mM Tris‐HCl, pH 7.5, and 50 mM NaCl), and then resuspended in 20 μl of reaction buffer (25 mM Tris‐HCl, pH 7.5, 50 mM NaCl, and 2 mM MgCl_2_) containing Fic‐1 and AntF, and further incubated at 30°C for 30 min. Then, 10 μl click‐chemistry master mix was added to each protein sample. Subsequent click chemistry, SDS‐PAGE separation, fluorescence imaging, and Coomassie brilliant blue staining were performed as described for the AMPylation assay.

### Bacterial growth analysis

To evaluate the effects of Fic‐1 on bacterial growth, *E. coli* BL21 (DE3) was transformed with either pET22b‐*fic‐1* or the *fic‐1* mutant construct. Transformants were screened on LB plates containing kanamycin. For liquid culture growth assays, colonies from freshly streaked LB plates were inoculated into LB broth and cultured overnight at 37°C. Cells were then harvested, washed twice with PBS, and resuspended to uniform density based on optical density measurements following appropriate dilutions. Equal‐density inocula were used to initiate cultures in 150 ml of LB broth. After a 30‐min incubation at 37°C, each culture was divided into three replicate subcultures and incubated in a shaker at 200 rpm. Bacterial growth was monitored by measuring OD_600_ at 2 h intervals. Growth curves were generated by plotting the mean OD_600_ values of the three biological replicates against the incubation time.

### Alignment of protein sequences

Protein sequences of Fic and its homologs were searched and retrieved from the UniProt Knowledgebase (https://www.uniprot.org/). These sequences were subsequently aligned using the ClustalW to analyze and highlight the conserved amino acid residues within the characteristic Fic domain.

### Statistical tests

Statistical analyses were conducted using GraphPad Prism 7.00 (GraphPad Software). All experimental data were presented as the mean ± standard error of the mean (SEM). Statistical significance was determined using Student's *t*‐test or one‐way analysis of variance (ANOVA), followed by Tukey's or Fisher's least significant difference post hoc tests. Significance levels are indicated as follows: *p < 0.05; **p < 0.01; ***p < 0.001; and ****p < 0.0001.

## AUTHOR CONTRIBUTIONS

**Furong Chen**: Conceptualization; data curation; formal analysis; funding acquisition; investigation; methodology; writing—original draft. **Liwei Guo**: Data curation; formal analysis; funding acquisition; investigation; methodology; visualization; writing—original draft. **Canhua Lu**: Funding acquisition; validation; writing—review and editing. **Wenjun Jiang**: Project administration; writing—review and editing. **Zhao‐Qing Luo**: Conceptualization; project administration; writing—review and editing. **Junfeng Liu**: Conceptualization; funding acquisition; project administration; supervision; writing—review and editing. **Li‐Qun Zhang**: Conceptualization; funding acquisition; project administration; supervision; writing—original draft; writing—review and editing.

## ETHICS STATEMENT

This study did not involve any human participants or animal subjects.

## CONFLICT OF INTERESTS

The authors declare no conflict of interests.

## Supporting information

Figure S1. Effect of Ni^2+^ ions on the enzymatic activities of AMPylation and deAMPylation. Figure S2. Ca^2+^ does not inhibit the AMPylation activity of Fic‐1 in the presence of Mg^2+^. Figure S3. Schematic view of inter‐residue interactions at the Fic‐1–AntF binding interface generated using Ligplot v.4.5.3. Residues forming hydrogen bonds are shown in red for Fic‐1 and in black for AntF. Figure S4. Interactions between Fic‐1 and assorted AntF mutant proteins (A, C), and between AntF and assorted Fic‐1 mutant proteins (B, D), assessed by bacterial two‐hybrid assays (A, B) and pull‐down experiments (C, D). Cells were grown overnight in LB medium and spotted on LB agar containing X‐gal. Plates were incubated for 2 d at 28°C and then imaged (A, B). The ZIP/ZIP interactions were used as a positive control and strains harboring empty plasmids were used as negative controls. Data shown are representative of three independent experiments. Glutathione beads conjugated to GST‐Fic‐1 were incubated with AntF or AntF^S24A^, followed by immunoblotting using an anti‐AntF antibody to detect pulled down proteins (C). Glutathione beads conjugated to GST‐AntF were incubated with Fic‐1 or Fic‐1^R146E^, followed by immunoblotting using an anti‐Fic‐1 antibody to detect bound proteins (D). Figure S5. Structure overlay of Fic‐1 in the Fic‐1–AntF complex. Figure S6 Effect of R146 in Fic‐1 on the enzymatic activities of deAMPylation. Figure S7. A proposed model illustrating environmental adaptation mediated by the AMPylation catalyzed by Fic‐1 and deAMPylation carried out by the Fic‐1–AntF complex. Figure S8. Dose‐dependent deAMPylation by the Fic‐1–AntF complex. Figure S9. Molecular electrostatic surface potentials of Fic‐1^R146E^ (A) and the Fic‐1–AntF^E28G^ complex (B). Figure S10. Comparison of the amino acid sequence of Fic‐1 with its homologs. Figure S11. Analytical HPLC chromatogram of AntF variants. Table S1. Strains and plasmids used in this study. Table S2. X‐ray crystallography data collection and refinement statistics. Table S3. Primers used in this study.

## Data Availability

Protein structure data have been deposited in the Protein Data Bank (Fic‐1–AntF: 8WT0, Fic‐1^H135A^: 8WSX). All other study data are included in the article.
