# Supplementary material for: The toxin–antitoxin complex Fic‐1–AntF functions as a deAMPylase that regulates the activity of DNA gyrase
Source: mLife. 2026 Jun 25;5(3):301–11. doi: 10.1002/mlf2.70085 (PMC13327609; doi:10.1002/mlf2.70085)
Supplement: Supplementary file 1 — Figure S1. Effect of Ni2+ ions on the enzymatic activities of AMPylation and deAMPylation. Figure S2. Ca2+ does not inhibit the AMPylation activity of Fic‐1 in the presence of Mg2+. Figure S3. Schematic view of inter‐residue interactions at the Fic‐1–AntF binding interface generated using Ligplot v.4.5.3. Residues forming hydrogen bonds are shown in red for Fic‐1 and in black for AntF. Figure S4. Interactions between Fic‐1 and assorted AntF mutant proteins (A, C), and between AntF and assorted Fic‐1 mutant proteins (B, D), assessed by bacterial two‐hybrid assays (A, B) and pull‐down experiments (C, D). Cells were grown overnight in LB medium and spotted on LB agar containing X‐gal. Plates were incubated for 2 d at 28°C and then imaged (A, B). The ZIP/ZIP interactions were used as a positive control and strains harboring empty plasmids were used as negative controls. Data shown are representative of three independent experiments. Glutathione beads conjugated to GST‐Fic‐1 were incubated with AntF or AntFS24A, followed by immunoblotting using an anti‐AntF antibody to detect pulled down proteins (C). Glutathione beads conjugated to GST‐AntF were incubated with Fic‐1 or Fic‐1R146E, followed by immunoblotting using an anti‐Fic‐1 antibody to detect bound proteins (D). Figure S5. Structure overlay of Fic‐1 in the Fic‐1–AntF complex. Figure S6 Effect of R146 in Fic‐1 on the enzymatic activities of deAMPylation. Figure S7. A proposed model illustrating environmental adaptation mediated by the AMPylation catalyzed by Fic‐1 and deAMPylation carried out by the Fic‐1–AntF complex. Figure S8. Dose‐dependent deAMPylation by the Fic‐1–AntF complex. Figure S9. Molecular electrostatic surface potentials of Fic‐1R146E (A) and the Fic‐1–AntFE28G complex (B). Figure S10. Comparison of the amino acid sequence of Fic‐1 with its homologs. Figure S11. Analytical HPLC chromatogram of AntF variants. Table S1. Strains and plasmids used in this study. Table S2. X‐ray crystallography data collect [file MLF2-5-301-s001.docx]

SUPPLEMENTARY FIGURES


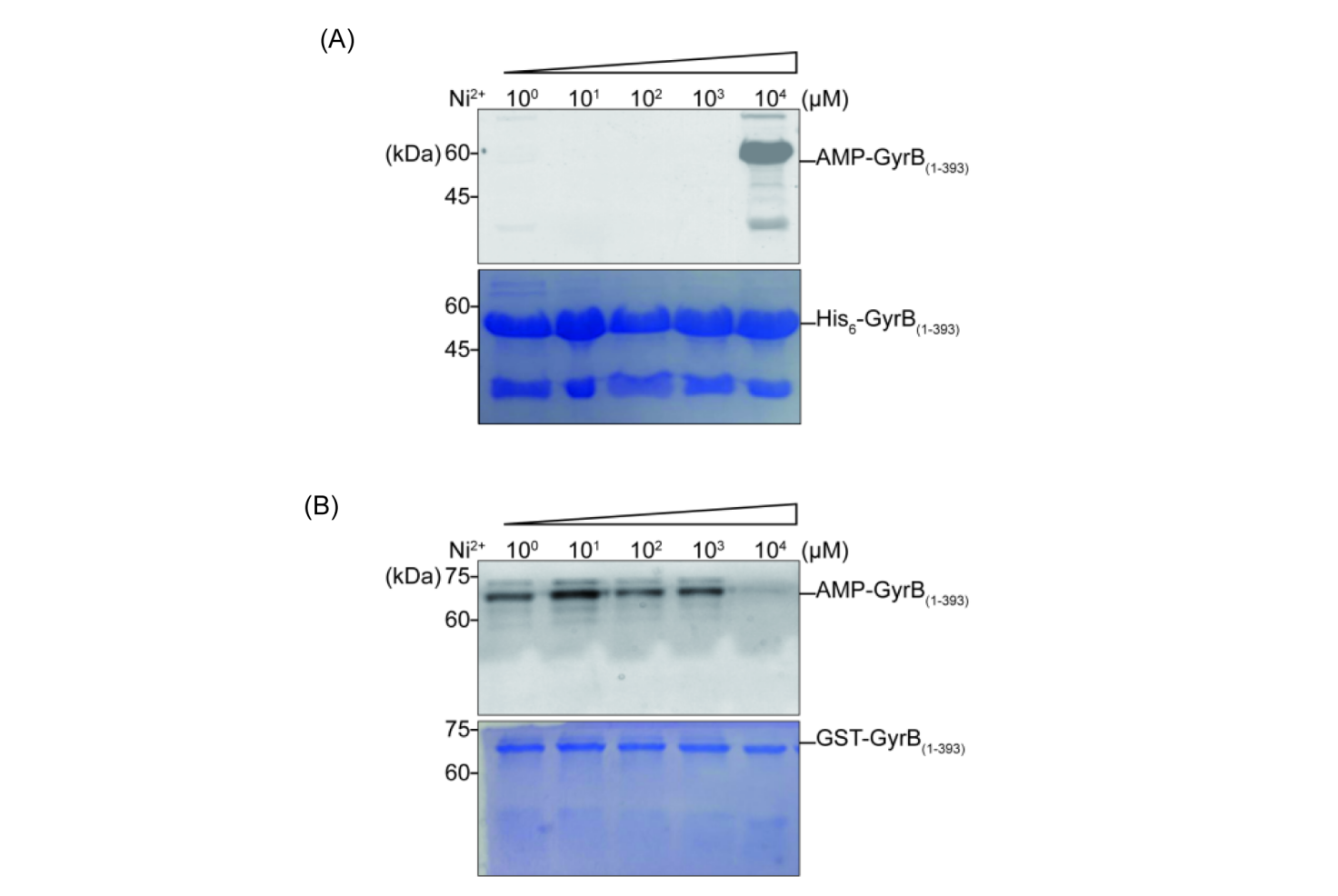


Figure S1 The effect of Ni^2+^ ions on the enzymatic activities of AMPylation and deAMPylation

(A) AMPylation reactions were carried out using N^6^pATP in the presence of varying concentrations of Ni^2+^ ions. (B) DeAMPylation of N^6^pAMP-GyrB_(1-393)_ by Fic-1 and AntF in the presence of different concentrations of Ni^2+^ ions. The reaction was conducted for 10 min at 30 °C. N^6^pAMP-GyrB_(1-393)_ was detected by fluorescence scanning (top panels in A and B), and the protein levels were confirmed by Coomassie blue staining (bottom panels in A and B).


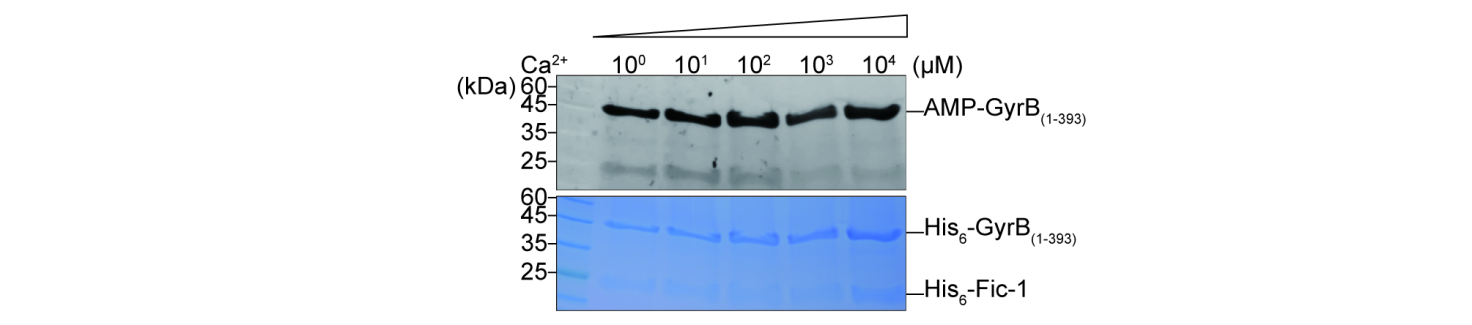


Figure S2. Ca^2+^ does not inhibit the AMPylation activity of Fic-1 in the presence of Mg^2+^.

AMPylation reaction was carried out in a fixed concentration of Mg^2+^ (1 mM) and increasing concentration of Ca^2+^ (1, 10, 100, 1000 μM or 10 mM). Reactions proceeded for 10 min at 30°C. N^6^pAMP-GyrB_(1-393)_ was detected by fluorescence scanning and protein levels were verified by Coomassie blue staining.


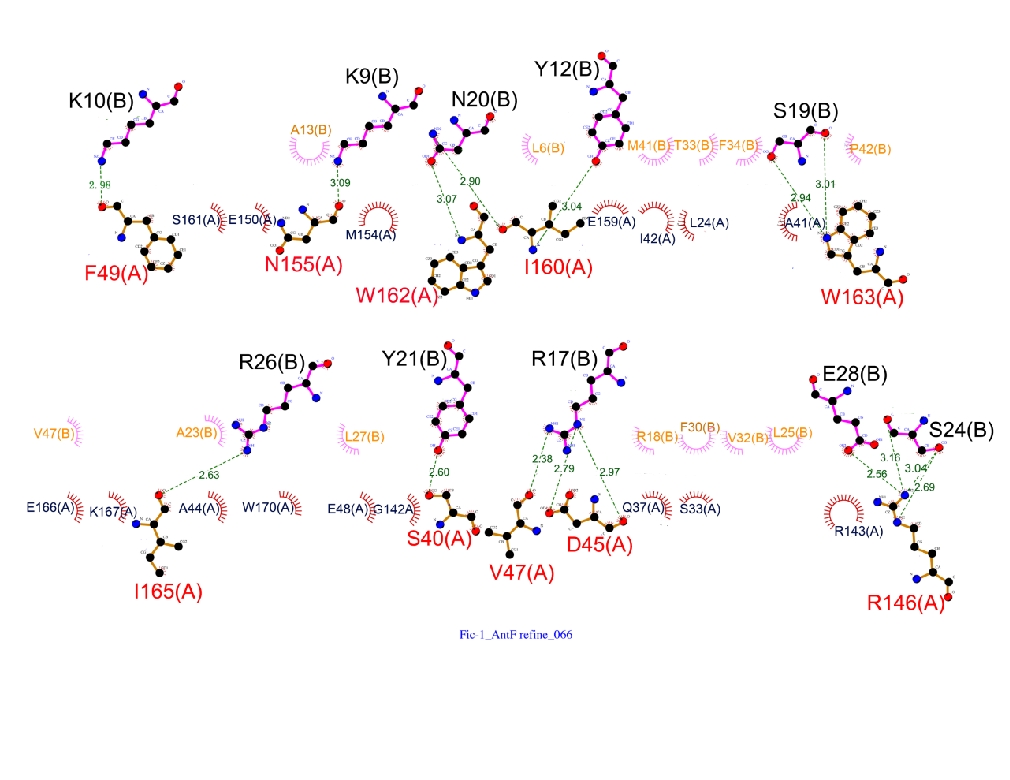


Figure S3. Schematic view of inter-residue interactions at the Fic-1-AntF binding interface generated using Ligplot v.4.5.3. Residues forming hydrogen bonds are shown in red for Fic-1 and in black for AntF.


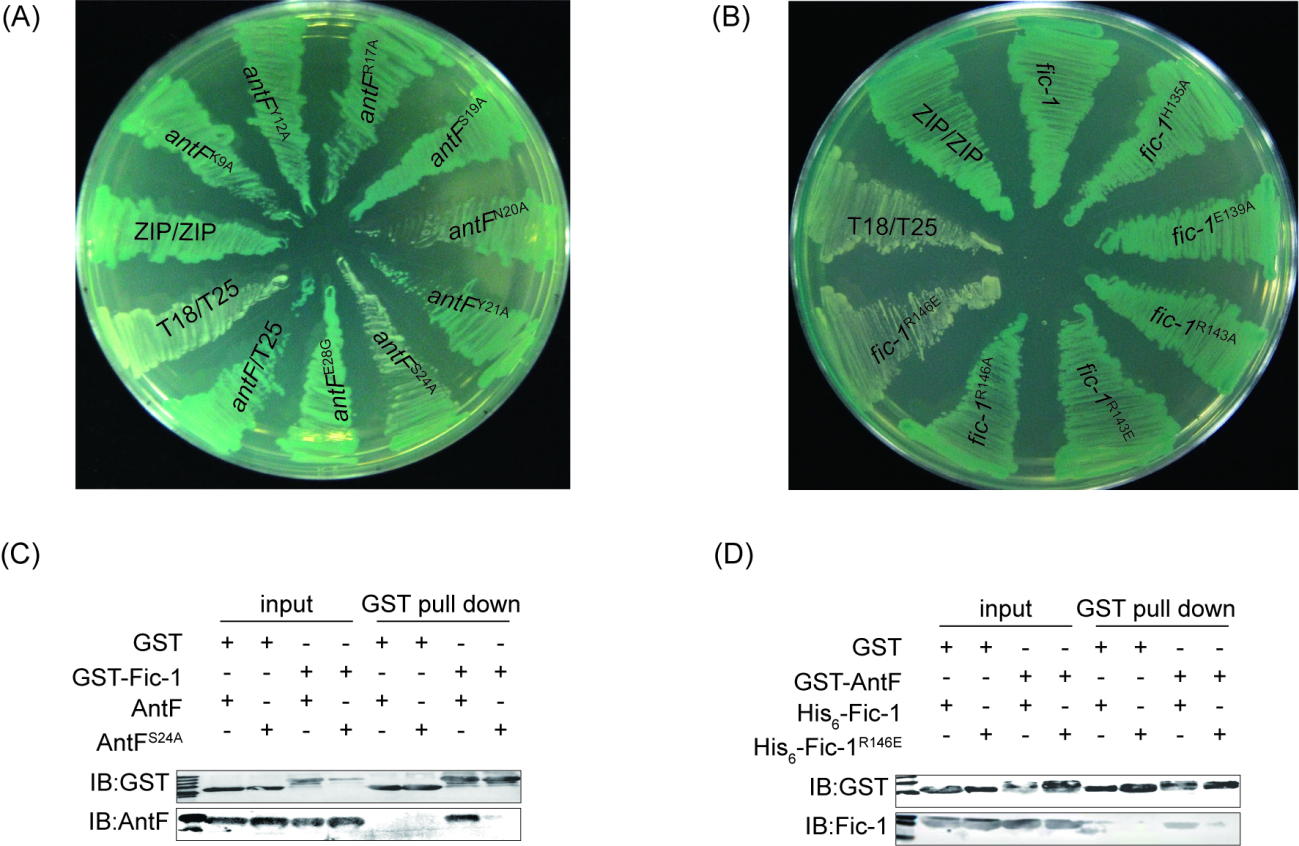


Figure S4. Interactions between Fic-1 and assorted AntF mutant proteins (A, C), and between AntF and assorted Fic-1 mutant proteins (B, D), assessed by bacterial two-hybrid assays (A, B) and the pull-down experiments (C, D). Cells were grown overnight in LB medium and spotted on LB agar containing X-gal. Plates were incubated for 2 d at 28°C and then imaged (A, B). The ZIP/ZIP interaction were used as a positive control, and strains harboring empty plasmids were used as negative controls. Data shown are representative of three independent experiments. Glutathione beads conjugated to GST-Fic-1 were incubated with AntF or AntF^S24A^, followed by immunoblotting using an anti-AntF antibody to detect pulled-down proteins (C). Glutathione beads conjugated to GST-AntF were incubated with Fic-1 or Fic-1^R146E^, followed by immunoblotting using an anti-Fic-1 antibody to detect bound proteins (D).


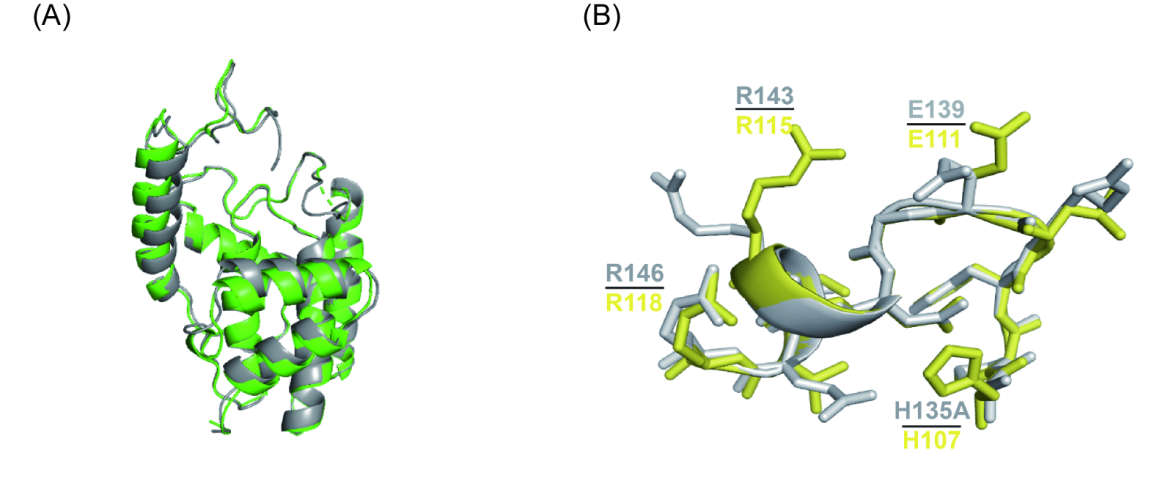


Figure S5. Structure overlay of Fic-1 in Fic-1-AntF complex.

1. Structure superposition of the Fic-1 within Fic-1-AntF complex (green) and the Fic-1^H135A^ (gray), shown as ribbon diagrams.

(B) Detailed view of the active site alignment between the FIC domain of Fic-1^H135A^ (grey) and NmFic (SE/AA) (yellow, PDB: 3SN9).


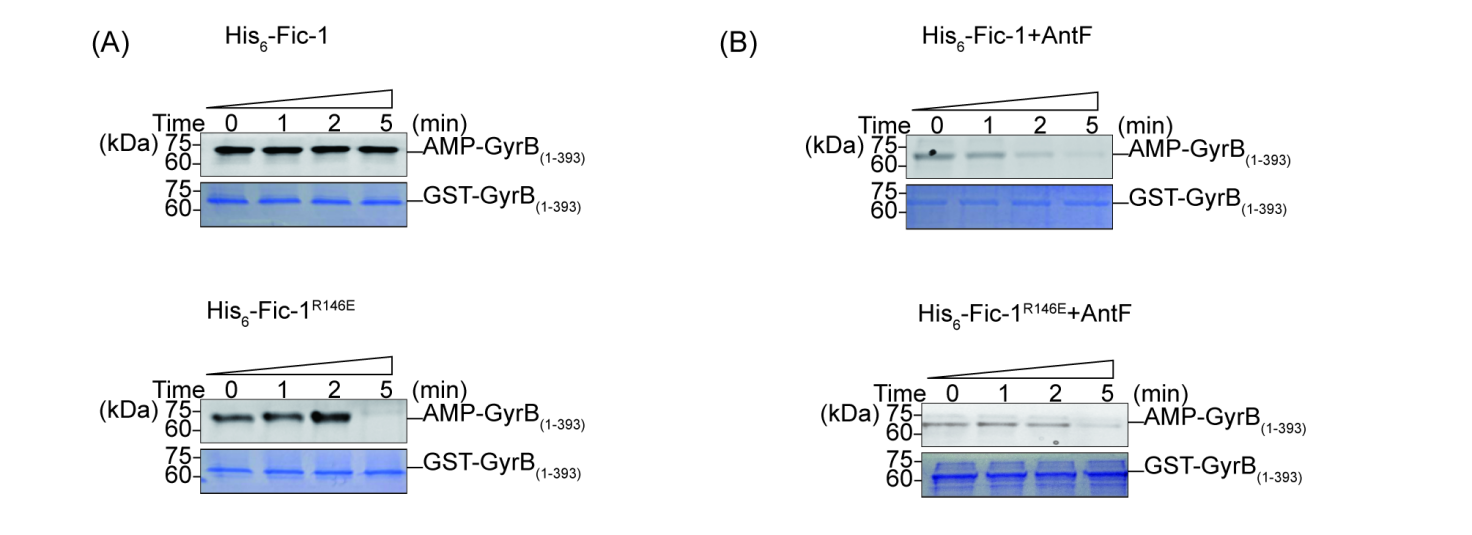


Figure S6 Effect of R146 in Fic-1 on the enzymatic activities of deAMPylation

(A) DeAMPylation of N^6^pAMP-GyrB _(1-393)_ by Fic-1. His_6_-Fic-1 or His_6_-Fic-1^R146E^ was added to reaction mixtures containing N^6^pAMP-GyrB_(1-393)_, and reactions were terminated at the indicated time points. (B) DeAMPylation of N^6^pAMP-GyrB _(1-393)_ by Fic-1 and AntF. His_6_-Fic-1 or His_6_-Fic-1^R146E^ was added into reaction mixtures containing N^6^pAMP-GyrB_(1-393)_ and AntF, and reactions were terminated at the indicated time points. N^6^pAMP-GyrB_(1-393)_ was detected by fluorescence scanning (top panels), and protein levels were assessed by Coomassie blue staining (bottom panels).


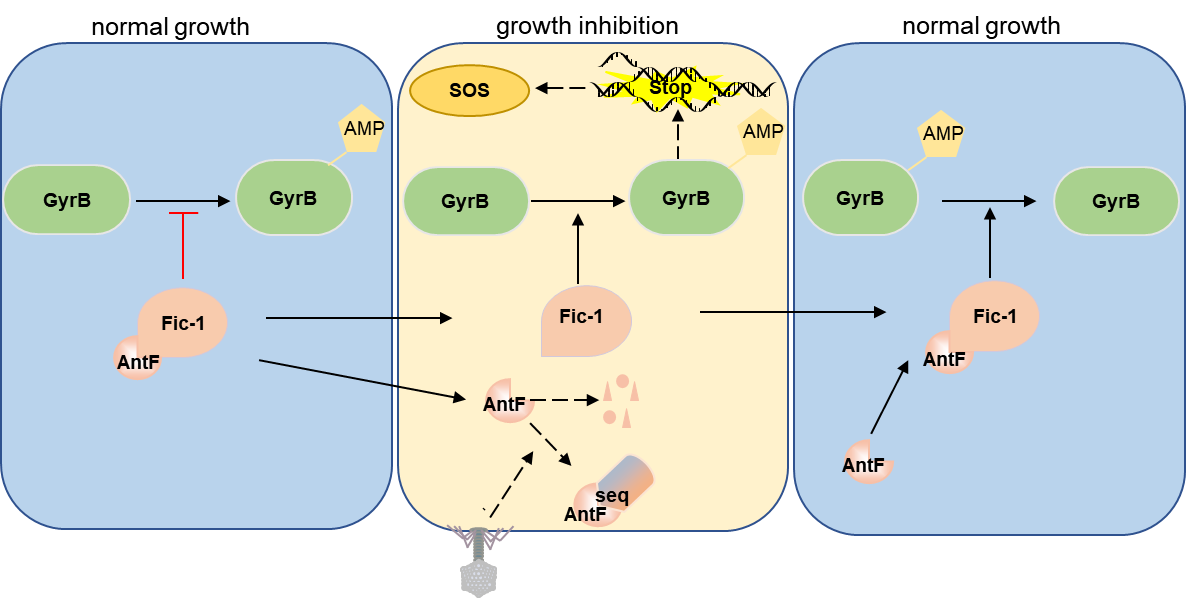
Figure S7. A proposed model illustrating environmental adaptation mediated by the AMPylation catalyzed by Fic-1 and deAMPylation carried out by the Fic-1-AntF complex.

Under favorable conditions, the complex formed by AntF and Fic-1 in *Pseudomonas bijieensis* 2P24 suppresses the AMPylation activity of Fic-1, enabling normal bacterial growth. However, under stressful conditions (such as exposure to antibiotics or bacteriophage infection), external stimuli trigger the degradation of the labile antitoxin AntF, mediated either by stress-induced proteases such as Lon or Clp, or by an unidentified high-affinity interacting factor, leading to the release of Fic-1. The liberated Fic-1 catalyzes the AMPylation of GyrB, resulting in its inactivation, inhibition of DNA replication, and suppression of bacterial growth (adaptive responses that facilitate survival during environmental stress). Upon restoration of optimal conditions and cessation of the inducing signal, AntF accumulates and reassociates with Fic-1 to form a functional complex that acts as a deAMPylase, efficiently removing the AMP moiety from modified GyrB, thereby restoring gyrase activity and permitting resumption of normal bacterial growth.


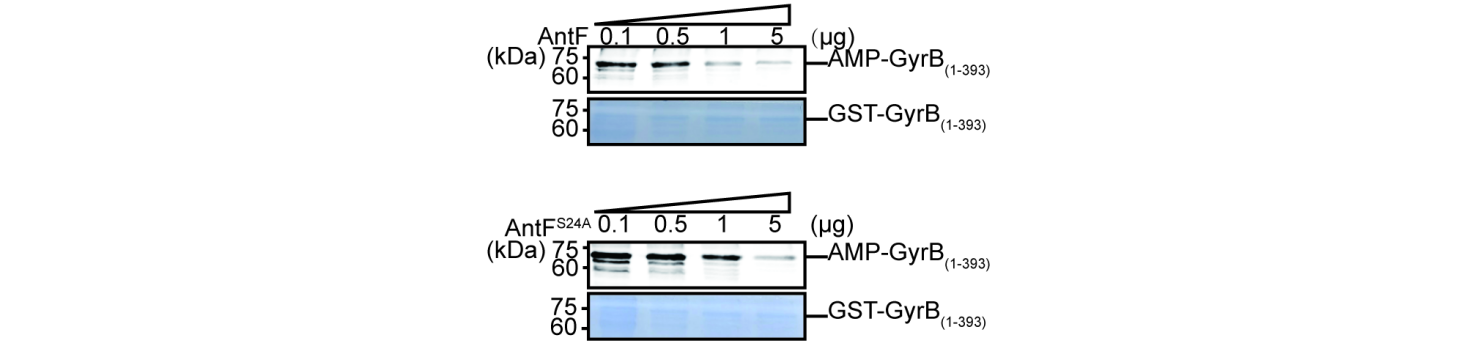


Figure S8. Dose-dependent deAMPylation by the Fic-1-AntF complex.

The reaction mixtures containing varying amounts of AntF (0.1, 0.5, 1, and 5 μg), His^6^-Fic-1 and N^6^pAMP-GyrB_(1-393)_ were incubated at 30 °C for 10 min. N^6^pAMP-GyrB_(1-393)_ was detected by fluorescence scanning (top panels), and protein levels were assessed by Coomassie blue staining (below panels). AntF^S24A^ was subjected to the same assay conditions.


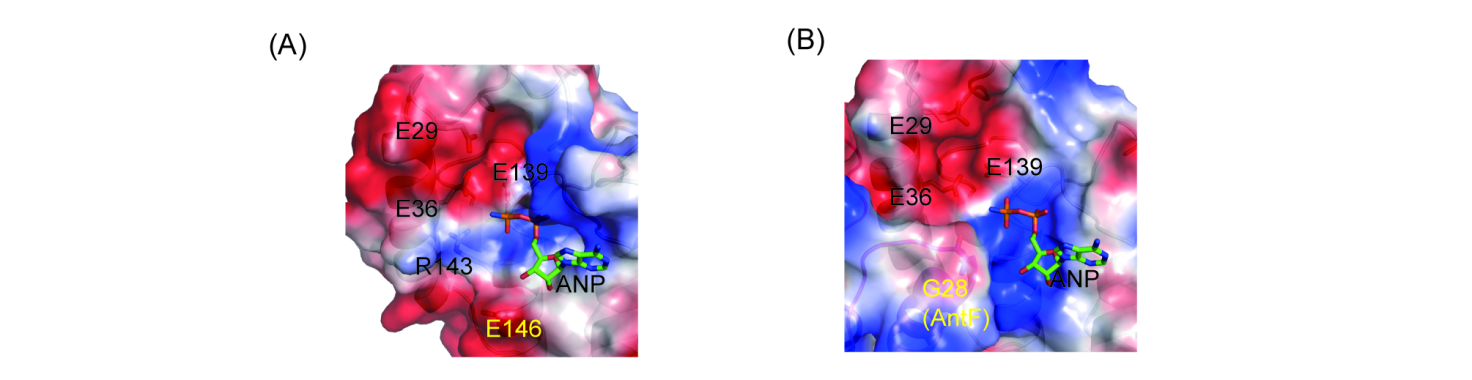


Figure S9. Molecular electrostatic surface potentials of Fic-1^R146E^ (A) and the Fic-1-AntF^E28G^ complex (B).

The structural model of Fic-1^R146E^ was generated using PyMOL & Coot based on the Fic-1^H135A^ structure. The structural model of the Fic-1-AntF^E28G^ complex was built using PyMOL & Coot based on the wild-type Fic-1-AntF structure. Positively and negatively charged electrostatic surfaces are colored blue and red, respectively; AntF is shown in purple*.*


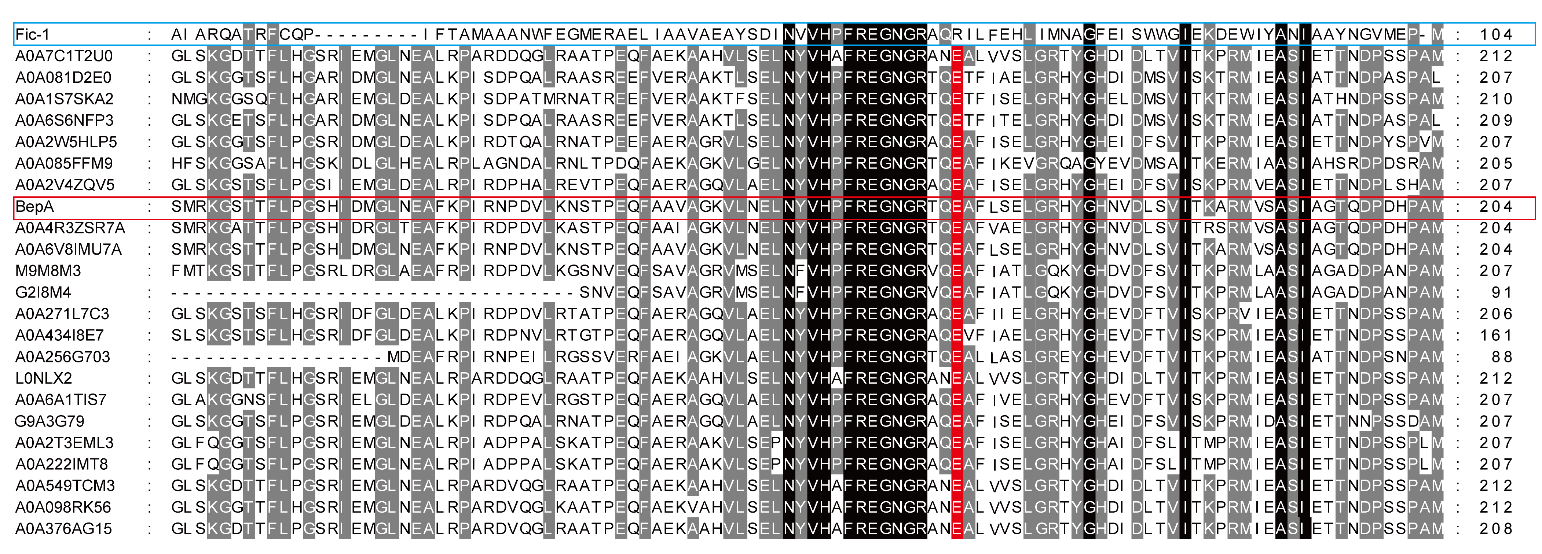
Figure S10. Comparison of the amino acid sequence of Fic-1 with its homologs.

Residues at position 146 are highlighted in red; glutamate at this position in Fic-1 is indicated. Sequence similarities are represented by varying shades of color. The blue box indicates the location of Fic-1 and red box marks the location of BepA-like protein from *Gluconobacter cerinus*.


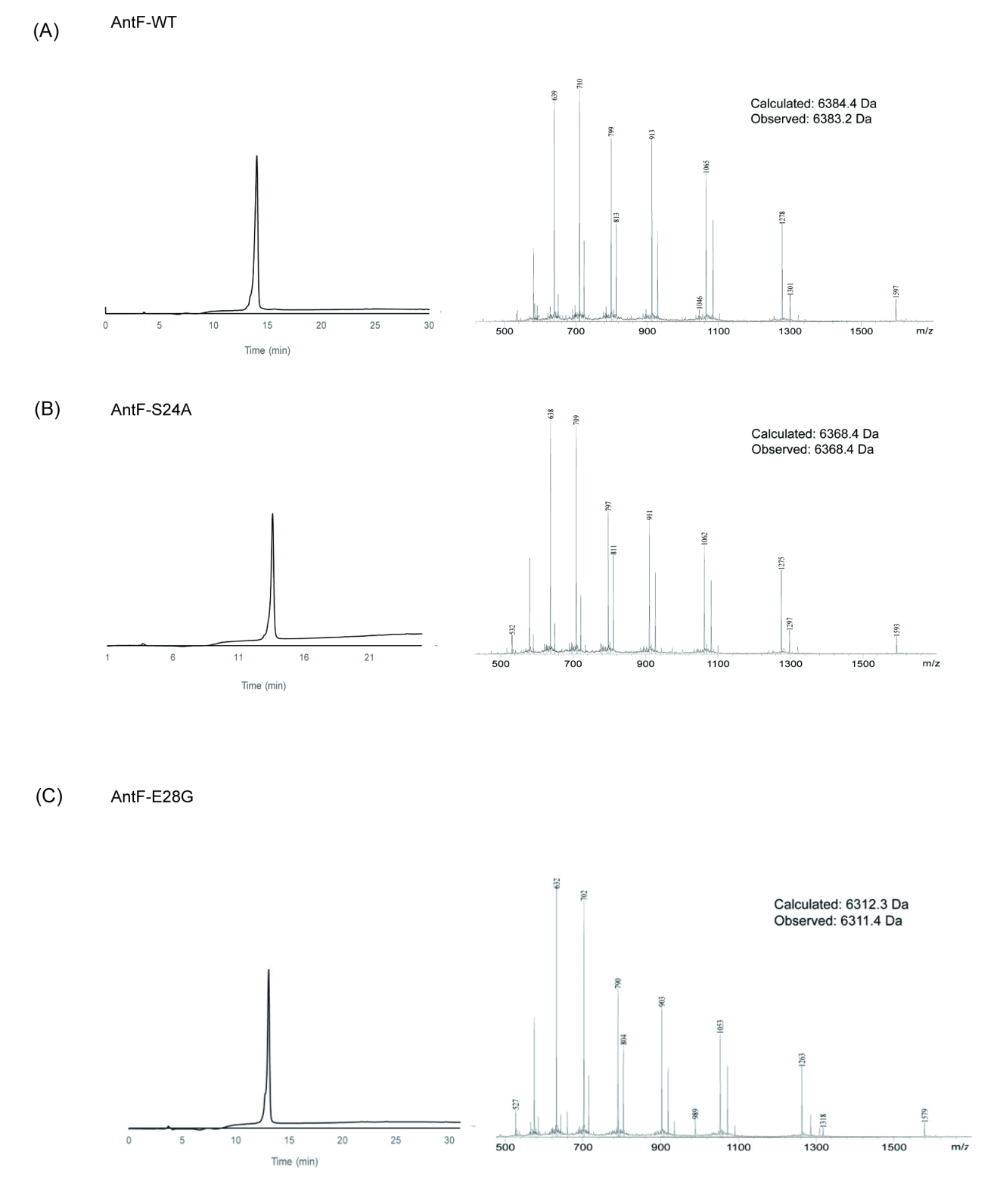
Figure S11. Analytical HPLC chromatogram of AntF variants.

Analytical HPLC chromatograms of the AntF-WT (A), AntF^S24A^ (B), and AntF^E28G^ (C) (λ=214 nm). Column: GL Sciences C4. Gradient: 20%-70% acetonitrile in water, both containing 0.1% trifluoroacetic acid (left panels). ESI-MS spectrum of the AntF-WT (A), AntF^S24A^ (B), and AntF^E28G^ (C) (right panels).

Table S1. Strains and plasmids used in this study

| Name | Relevant characteristics | References or sources |
| --- | --- | --- |
| Strains |  |  |
| *Escherichia coli* DH5α | F-, *φ80dlacZΔM15*, *Δ*(*lacZYA-argF*)*U169*, *deoR*, *recA1*, *endA1*, *hsdR17*(*rk-*, *mk+*), *phoA*, *supE44*, *λ-*, *thi-1*, *gyrA96, relA1.* | 1 |
| *E. coli* BTH101 | Str^R^; F-, *cya-99*, *araD139, galE15, galK16, rpsL1*, *hsdR2, mcrA1, mcrB1.* | 2 |
| *E. coli* BL21(DE3) | F-, *ompT*, *hsdS*(*rBB-mB-*), *gal*, *dcm*(*DE3*)*.* | Novagen |
| *Pseudomonas bijieensis* 2P24 | Amp^R^; wide type | 3 |
| Vectors |  |  |
| pET22b (+) | Amp^R^; expression vector | Novagen |
| pET-SUMO | Kan^R^; expression vector | Champion |
| pGEX-6P1 | Amp^R^; expression of GST-fusion protein | Our collection |
| pUT18C | Amp^R^; Encodes amino acids 225 to 399 of CyaA | 2 |
| pKT25 | Kan^R^;Encodes the first 224 amino acids of CyaA | 2 |
| pACYC Duet-1 | Cm^r^; Expression vector | Novagen |

|  |
| --- |

|  |
| --- |

Table S2. X‐ray crystallography data collection and refinement statistics

|  | Fic-1^H135A^ | Fic-1-AntF |
| --- | --- | --- |
| Wavelength (Å) | 0.9795 | 0.9795 |
| Resolution range (Å) | 42.99-2.57 (2.662-2.57) | 48.41-2.5 (2.589-2.5) |
| Space group | P1_2_11 | P6_3_22 |
| Unit cell | 80.918, 85.988, 136.613,  90, 93.691, 90 | 107.026, 107.026, 113.529,  90, 90, 120 |
| Total reflections | 387678 (42188) | 264259 (24903) |
| Unique reflections | 59544 (5901) | 13820 (1324) |
| Multiplicity | 6.5 (7.1) | 19.1 (18.7) |
| Completeness (%) | 99.39 (99.88) | 99.65 (99.40) |
| Mean I/sigma(I) | 11.80 (2.23) | 23.02 (3.07) |
| Wilson B-factor (Å^2^) | 53.52 | 57.26 |
| R-merge | 0.09639 (0.7331) | 0.1042 (1.187) |
| R_meas_ | 0.1055 (0.7906) | 0.1071 (1.221) |
| R_pim_ | 0.04206 (0.2939) | 0.02439 (0.2808) |
| CC_1/2_ | 0.997 (0.879) | 1 (0.967) |
| CC* | 0.999 (0.967) | 1 (0.991) |
| Reflections used in refinement | 59463 (5896) | 13776 (1325) |
| Reflections used for R_free_ | 2991 (310) | 708 (57) |
| R_work_ | 0.2477 (0.3435) | 0.2117 (0.2934) |
| R_free_ | 0.2797 (0.3420) | 0.2685 (0.2934) |
| CC(work) | 0.927 (0.801) | 0.954 (0.951) |
| CC(free) | 0.879 (0.714) | 0.923 (0.878) |
| Number of non-hydrogen atoms | 12311 | 1856 |
| -macromolecules | 12156 | 1801 |
| -ligands | - | 33 |
| -solvent | 155 | 22 |
| Protein residues | 1528 | 225 |
| RMS(bonds) (Å) | 0.009 | 0.005 |
| RMS(angles)(°) | 1.49 | 0.99 |
| Ramachandran favored (%) | 99.21 | 99.09 |
| Ramachandran allowed (%) | 0.79 | 0.91 |
| Ramachandran outliers (%) | 0.00 | 0.00 |
| Rotamer outliers (%) | 0.16 | 0.55 |
| Clashscore | 11.51 | 5.86 |
| Average B-factor(Å^2^) | 66.08 | 79.58 |
| macromolecules | 66.26 | 79.23 |
| ligands | - | 110.87 |
| solvent | 52.57 | 60.80 |
| Number of TLS groups | 47 | 5 |

Numbers in phenix.table_one has been used to calculated the table.

*R*merge = 100 × Σ*_hkl_* |Σ_i_ |*I_i_*(*hkl*) −〈*I*(*hkl*)〉| / Σ*_hkl_* |Σ_i_*I_i_*(*hkl*), where *I*(*hkl*)〉is the mean value of *I*(*hkl*).

*R*work = 100 × Σ*_hkl_* ||*F*_o_| − |*F*_c_|| / Σ*_hkl_* |*F*_o_|, where *F*o and *F*c the observed and calculated structure factors,

respectively.

*R*_free_ is calculated as for *R*_work_, but for the test set comprising 5% reflections not used in refinement.

Table S3. Primers used in this study

| Primer | Sequence（5’-3’） |
| --- | --- |
| AntF^E28G^-Forward | CCAGCCTTCGCCTGGGAGGCTTCAAAGTGAC |
| AntF^E28G^-Reverse | GTCACTTTGAAGCCTCCCAGGCGAAGGCTGG |
| AntF-1-XbaⅠ-Forward | ATTCTAGAGGGCAATGTCAGCCTTGAAA |
| AntF-30-KpnⅠ-Reverse | ATGGTACCGAAGCCTTCCAGGCGAAGGC |
| AntF-31-XbaⅠ-Forward | ATTCTAGAGAAAGTGACCTTCGCGGACGG |
| AntF-56-KpnⅠ-Reverse | ATGGTACCTCAGGTTCGGGTCTGGGTGA |
| pUT18C-Forward | TGGAAACGGTGCCGGCGTCA |
| pUT18C-Reverse | CTGAGAGTGCACCATATTACTT |
| AntF^E28G^-Forward | CCAGCCTTCGCCTGGGAGGCTTCAAAGTGAC |
| AntF^E28G^-Reverse | GTCACTTTGAAGCCTCCCAGGCGAAGGCTGG |
| GST-His-GyrB-Forward | CACCACCACCACCACCACGGATCCTTGAGCGAAGAA |
| GST-His-GyrB-Reverse | GTGGTGGTGGTGGTGGTGCAGGGGCCCCTGGAACAGAA |
| pGEX-6P-1-Forward | AAGTTCTGTTCCAGGGGCCC |
| pGEX-6P-1-Reverse | GAAACGCGCGAGGCAGATC |
| pET-SUMO-Forward | GAAAACCTGTACTTCCAATCCA |
| pET-SUMO-Reverse | TGTTAGCAGCCGGATCTCAGT |
| Fic-1^R146A^-Forward | CAATGGCCGTGCGCAAGCCATCCTATTTGAACACC |
| Fic-1^R146A^-Reverse | GGTGTTCAAATAGGATGGCTTGCGCACGGCCATTG |
| Fic-1^R146E^-Forward | AAGGCAATGGCCGTGCGCAAGAGATCCTATTTGAACACCTC |
| Fic-1^R146E^-Reverse | GAGGTGTTCAAATAGGATCTCTTGCGCACGGCCATTGCCTT |
| Fic-1^R143A^-Forward | GCGAAGGCAATGGCGCTGCGCAACGCATCC |
| Fic-1^R143A^-Reverse | GGATGCGTTGCGCAGCGCCATTGCCTTCGC |
| Fic-1^R143E^-Forward | CCGCGAAGGCAATGGCGAGGCGCAACGCATCCTAT |
| Fic-1R^143E^-Reverse | ATAGGATGCGTTGCGCCTCGCCATTGCCTTCGCGG |
| Fic-1^E139A^-Forward | ACCCCTTCCGCGCAGGCAATGGCCG |
| Fic-1^E139A^-Reverse | CGGCCATTGCCTGCGCGGAAGGGGT |
| Fic-1^H135A^-Forward | CGCGGAAGGGGGCTACGACGTTGATGTCGGAATA |
| Fic-1^H135A^-Reverse | TATTCCGACATCAACGTCGTAGCCCCCTTCCGCG |
| AntF^S24A^-Forward | CCAACTACGCGGCCGCCCTTCGCCTGGAAG |
| AntF^S24A^-Reverse | CTTCCAGGCGAAGGGCGGCCGCGTAGTTGG |
| pUT18C-F | TGGAAACGGTGCCGGCGTCA |
| pUT18C-R | CTGAGAGTGCACCATATTACTT |
| pET22b-F | CACCACCACCACCACCACTG |
| pET22b-R | GGCCATCGCCGGCTGGGCAGCG |
| GST-His-GyrB-F | CACCACCACCACCACCACGGATCCTTGAGCGAAGAA |
| GST-His-GyrB-R | GTGGTGGTGGTGGTGGTGCAGGGGCCCCTGGAACAGAA |
| pGEX-6P-1-F | AAGTTCTGTTCCAGGGGCCC |
| pGEX-6P-1-R | GAAACGCGCGAGGCAGATC |
| pET-SUMO-F | GAAAACCTGTACTTCCAATCCA |
| pET-SUMO-R | TGTTAGCAGCCGGATCTCAGT |
| Fic-1-F | GCCCAGCCGGCGATGGCCATGCCTGACAAATATGGG |
| Fic-1-R | GTGGTGGTGGTGGTGGTGAGCTTGAATCGCCTGCCC |
| GST-His-GyrB-F | CACCACCACCACCACCACGGATCCTTGAGCGAAGAA |
| GST-His-GyrB-R | GTGGTGGTGGTGGTGGTGCAGGGGCCCCTGGAACAGAA |

**References**

1. Hanahan D. Studies on transformation of *Escherichia coli* with plasmids. *J Mol Biol.* 1983;166:557-580.
2. Karimova G, Pidoux J, Ullmann A, Ladant D. A bacterial two-hybrid system based on a reconstituted signal transduction pathway. *Proc Natl Acad Sci U S A.* 1998;95:5752-5756.
3. Wei HL, Zhang LQ. Quorum-sensing system influences root colonization and biological control ability in *Pseudomonas fluorescens* 2P24. *Antonie Van Leeuwenhoek.* 2006;89:267-280.
